# Storage stability and shelf‐life of soymilk obtained via repeated boiling and filtering: A predictive model

**DOI:** 10.1002/fsn3.3893

**Published:** 2023-12-15

**Authors:** Liu Fan, Yitong Duan, Zhanrui Huang, Dan Zhao, Liangzhong Zhao, Wanying He, Xuejiao Zhang, Ming Li, Yingyi Lin, Yu Chen

**Affiliations:** ^1^ College of Food and Chemical Engineering, Hunan Provincial Key Laboratory of Soybean Products Processing and Safety Control Shaoyang University Shaoyang Hunan China; ^2^ Kangdeli Intelligent Technology (Zhejiang) CO., LTD Jiaxing China; ^3^ Hunan Genda Fiber Tech Mechanical CO., LTD Changsha China

**Keywords:** repeated boiling‐to‐filtering method, shelf‐life, soymilk, storage stability

## Abstract

This study investigated the effects of different processing methods on the quality and nutrition of soymilk, as well as the changes in storage stability (centrifugal sedimentation rate (CSR), viscosity, and particle size) and shelf‐life of soymilk at different storage temperatures (25°C, 35°C, 45°C, and 55°C). Results showed that soymilk processed via the repeated boiling‐to‐filtering method (RBFM) exhibited the highest protein content (3.89 g/100 g), carbohydrate content (1.27 g/100 g), and stability coefficient (0.950). The CSR and particle size of RBFM soymilk increased gradually during storage at different temperatures, while the viscosity and sensory score decreased. The correlation between the CSR and the sensory score of RBFM soymilk was the highest (*R* ^2^ = .9868). The CSR was selected as the key indicator to predict the shelf‐life of RBFM soymilk. The average residual variation in RBFM soymilk shelf‐life based on the predictive model was 10.78%, indicating the strong accuracy of the model for predicting the shelf‐life of RBFM soymilk stored at temperatures ranging from 25–45°C. This study provides a theoretical basis and technological support for the development, transportation, and storage of soymilk and soymilk beverage products.

## Graphical abstract

Repeated boiling‐to‐filtering method (RBFM) soymilk exhibited the highest nutrient content and physical properties than FBM and BFM soymilk. The sensory score and stability indices of RBFM soymilk changed significantly during storage at different temperatures. A shelf‐life prediction model was constructed with CSR as the key indicator, which can be used to predict the shelf‐life of RBFM soymilk stored at temperatures ranging from 25 to 45°C.

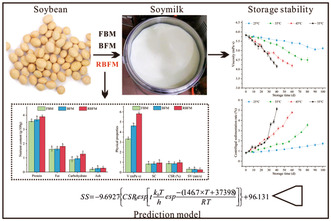

## 1 INTRODUCTION

Soybean is rich in proteins, vitamins, inorganic salts, minerals, isoflavones, and other nutrients. Its protein content is two‐ to three‐fold higher than that of eggs and one‐ to two‐fold higher than that of meat, and therefore it is known as “the warehouse of high‐quality protein” (Cabanos et al., 2021; Friedman & Brandon, 2001; Li et al., 2023). Soymilk is obtained from soybean through complex processes. Soymilk contains different proportions of nutritional components present in soybean (Ju et al., 2021). In recent years, soymilk has become popular because of its rich nutritional value and health benefits. It is easily digested and absorbed, and therefore plays an important role in human nutrition (Atuahene et al., 2018; Hasan et al., 2023). A large number of studies have shown that long‐term consumption of soymilk reduces the occurrence of chronic conditions such as constipation, hypertension, hyperlipidemia, cardiovascular, and cerebrovascular sclerosis (Omonia & Aluko, 2005; Singh et al., 2008; Xiao, 2008).

Recent studies highlighting the nutritional value and health benefits of soymilk have drawn the attention of researchers to changes in the quality of soymilk and its flavor (Kaharso et al., 2021; Wang et al., 2021; Xu et al., 2022). The soymilk products are graded based on the quality of the soymilk, which is an important economic and safety indicator. The quality of soymilk is evaluated based on multiple parameters, such as particle size, viscosity, and centrifugal sedimentation rate (CSR; Kamizake et al., 2016; Mu et al., 2022; Zuo et al., 2016). Therefore, monitoring the quality of soymilk is key to the development of soybean products industrially. Soymilk processing technology is complex and diverse, and includes soaking, grinding, boiling, filtering, and other methods (Huang et al., 2022; Kumar et al., 2021; Zhang et al., 2021). Different pulping methods not only play a decisive role in the extraction of nutrients and sensory scores but also have a significant impact on the stability of soymilk (Nik et al., 2009; Yu et al., 2015). Three methods have been used to produce soymilk based on boiling and filtering: filtering‐to‐boiling method (FBM), boiling‐to‐filtering method (BFM), and repeated boiling‐to‐filtering method (RBFM; Huang et al., 2022).

Several studies reported changes in the quality of soymilk under the different methods. Li et al. (2012) found that the soybean flavor components of BFM soymilk were higher than in FBM soymilk, and the beany flavor compounds were lower than in FBM soymilk, indicating that BFM was more suitable for processing soymilk products with prominent soybean flavor. Kyoko et al. (2007) showed that the relative levels of protein and viscosity were higher in soymilk generated via BFM, while the pH value was lower. The BFM yielded significantly fewer liposomes. We reported previously that the tofu produced via RBFM has better flavor, higher nutritional content, and the highest stability than that generated via FBM (Huang et al., 2022). Therefore, further studies are needed to investigate the changes in storage stability of soymilk produced via RBFM and establish a model to predict its shelf‐life.

In this study, FBM, BFM, and RBFM were used to produce soymilk. The changes in sensory scores, nutritional composition, and physical properties were measured. In addition, accelerated storage tests of soymilk were conducted at different temperatures (25°C, 35°C, 45°C, and 55°C) after ultra‐high‐temperature instant sterilization. Changes in sensory scores, CSR, viscosity, and particle size of soymilk at different storage temperatures were measured. The Arrhenius equation was used to analyze the correlation between soymilk stability indicators and changes in soymilk quality at different storage temperatures. A predictive model for the shelf‐life of soymilk was established and validated. This study aims to provide effective technical support and a theoretical basis for the development, transportation, and storage of soymilk and its products.

## 2 MATERIALS AND METHODS

### 2.1 Materials

Canadian non‐GMO soybeans (protein content 38%) were purchased from Wanyue Import and Export Trade Co. Ltd.

### 2.2 Preparation of soymilk

**FIGURE 1:**
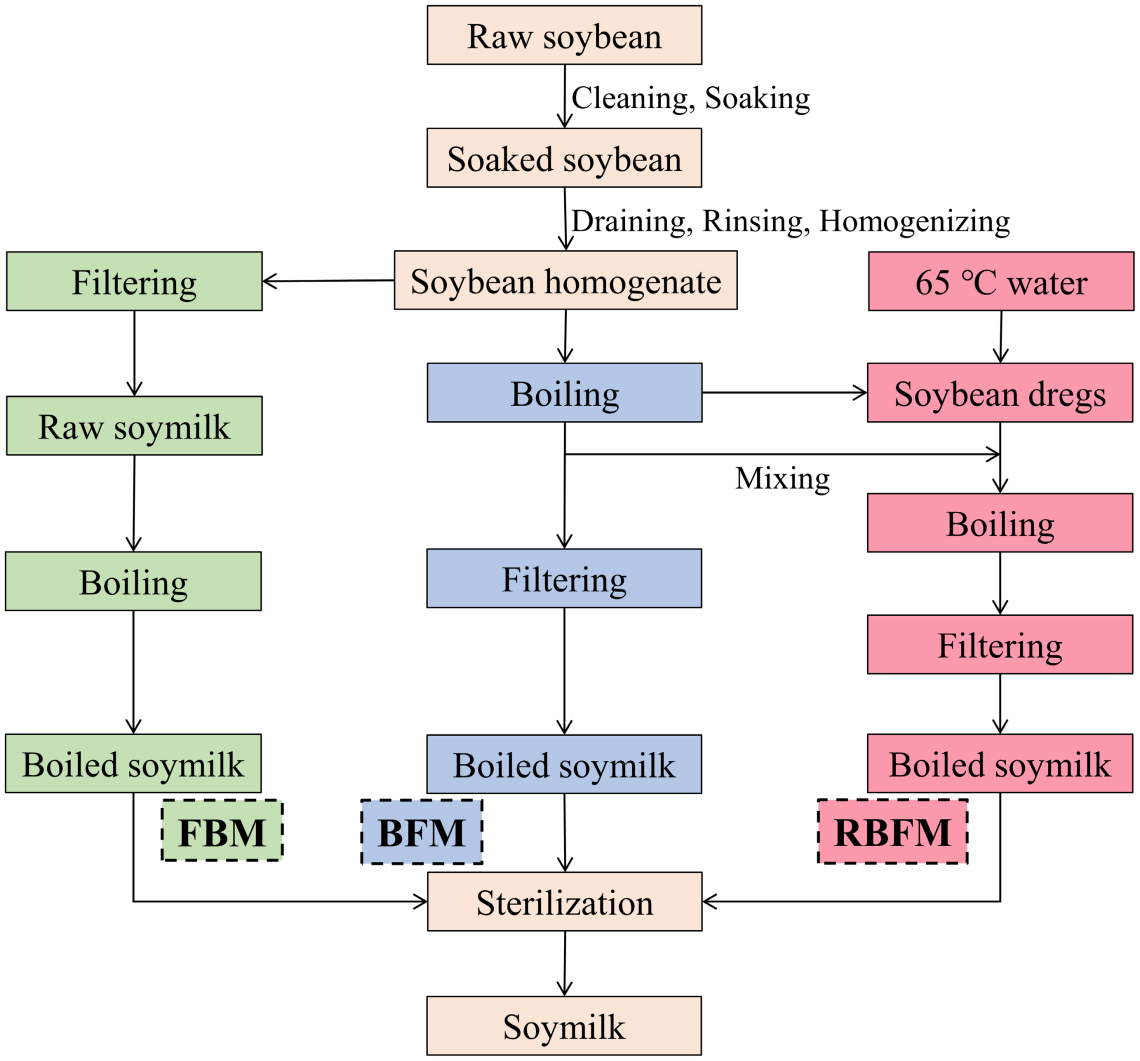
Flow chart outlining soymilk production. FBM, BFM, and RBFM refer to the filtering‐to‐boiling method, the boiling‐to‐filtering method, and the repeated boiling‐to‐filtering method, respectively. BFM, boiling‐to‐filtering method; FBM, filtering‐to‐boiling method; RBFM, repeated boiling‐to‐filtering method.

The soymilk was produced by FBM, BFM, and RBFM using the following steps, as illustrated in Figure 1.

Soaking: The soybeans were cleaned twice. Dry soybeans (5 kg) were soaked at 25°C for 8 h in a 1:3 soybean–water ratio. The weight of wet soybeans was approximately 10–11 kg.

Homogenizing: The soaked soybeans were homogenized with water (1:3.5 soybean–water ratio) in a soymilk grinder (SJJ‐20, Kangdeli Machinery Equipment Manufacturing Co. Ltd.).

Boiling: The raw soymilk was boiled to 105°C using soymilk‐integrated equipment (MZJJ‐1, Kangdeli Machinery Equipment Manufacturing Co., Ltd.). The temperature and pressure were maintained for 5 min to obtain soymilk A.

Filtering: A 74‐μm filter cloth was used for filtration.

Washing of soybean dregs: Water at 65°C was used to wash the soybean dregs at a ratio of 2:1.

Sterilization: Warm water was added to boiled soymilk to a constant volume of 45 L. The soymilk was sterilized at 139°C for 10 s at ultra‐high temperatures in an instantaneous sterilization machine (NHSY‐1, Shanghai Nanhua Heat Exchanger Manufacturing Co., Ltd.).

### 2.3 Accelerated storage test (accelerated destructive test)

Soymilk was filled aseptically in 250‐mL sterile bottles, and 6 of the soymilk was used as the control. Accelerated storage was tested by incubating 240 bottles at 25°C, 35°C, 45°C, and 55°C in a constant temperature incubator, and 60 bottles were tested at each temperature, including 10 groups of samples at each temperature and 6 parallel samples for each group. Soymilk samples (6 bottles) were randomly selected from incubators at 25°C, 35°C, 45°C, and 55°C for sensory evaluation and storage stability (CSR, viscosity, and particle size) testing at intervals of 4, 6, 8, and 10 days, respectively.

### 2.4 Sensory evaluation

The sensory evaluation (color, flavor, taste, and stability) of soymilk was conducted by trained panelists (10 females, 10 males, all in the ages ranging from 25 to 30 years) according to the methods described by Li, He, et al. (2022) and Li, Wan, et al. (2022). The soymilk samples were evaluated in triplicate by each panelist. Soymilk samples were separately packed in 50‐mL odorless transparent plastic cups, and then coded and evaluated using a randomized design. The evaluation criteria are shown in Table 1.

**TABLE 1:** Criteria for sensory evaluation of soymilk.

Indices	Criteria		
Color (20)	Milky white (17–20)	Light milky yellow (10–16)	Yellowish, other colors available (<10)
Flavor (30)	Rich bean aroma, no peculiar smell (25–30)	Light bean aroma, slightly beany flavor (15–24)	Strong beany flavor, with a bitter or other unpleasant flavor (<15)
Taste (30)	Delicate, silky, without obvious graininess (25–30)	Slight roughness, slight graininess (15–24)	Roughness, obvious graininess (<15)
Stability (20)	No fat floating or sedimentation (17–20)	Small amounts of fat floating or sedimentation (10–16)	Excessive fat floating or sedimentation (<10)

### 2.5 Nutritional composition of soymilk

The ash content of the soymilk sample was determined via the constant weight method (Huang, Sun, et al., 2021). Additionally, the protein content was determined via the Kjeldahl method (protein conversion factor: 6.25) and the fat content was determined via ether extraction (Shin et al., 2015).

Total carbohydrates, including crude fiber, were determined using the phenol–sulfuric acid method (Shin et al., 2015), with phenol solution (0.5 g/L) as the phenolic compound and D‐glucose as the standard sugar source (Baishakhi et al., 2022).

### 2.6 Determination of physical properties and storage stability indices of soymilk

#### 2.6.1 Viscosity

The viscosity of soymilk was measured with a viscometer (NDJ‐5S, Shanghai Pingxuan Scientific Instrument Co., Ltd.; Shimoyamada et al., 2019). The rotor was “0” (capable of measuring low viscosity to 0.1 mPa·s), with a speed of 60 r/min and a measurement temperature of 25°C.

#### 2.6.2 Particle size

The particle size distribution range and average particle size of soybean milk were measured with a laser particle size analyzer (WJL‐628, AMTETEK Inc; Luo, 2009). Distilled water was used as the dispersion medium. The refractive index of the real part was 1.76, while that of the imaginary part was 0.05. The ideal shading ratio was 1:2. The refractive index of the medium was 1.33.

#### 2.6.3 Centrifugal sedimentation rate

A 5 mL soymilk sample in a 10 mL centrifuge tube was centrifuged at 4192.5 × g for 10 min. The supernatant was discarded. The inverted tube was drained and weighed (Huo et al., 2023). Each experiment was performed in triplicate. The CSR was calculated as follows:

$$w_{1}=\left(\frac{m_{2}-m_{1}}{m_{0}}\right)\times 100,$$

In the above formula, *w*_1_ is the centrifugal sedimentation rate, %; *m*_0_ is the sample mass, mg; *m*_1_ is the mass of the centrifuge tube, mg; and *m*_2_ represents the mass of the centrifuge tube after discarding the supernatant, mg.

#### 2.6.4 Stability coefficient

A 5 mL sample of soybean milk was diluted 40‐fold with deionized water and centrifuged at 2683.2 × g for 5 min (Zhang et al., 2017). The absorbance of the sample before and after centrifugation was measured at 785 nm with an UV spectrophotometer (UV‐1780, Shimadzu). The stability coefficient was calculated as follows:

$$R=\frac{A_{2}}{A_{1}}$$

In the above formula, *R* is the stability coefficient; *A*_1_ denotes the absorbance of the sample before centrifugation; and *A*_2_ is the absorbance of the supernatant after centrifugation.

#### 2.6.5 Sedimentation velocity

According to the particle size, viscosity, and density of soymilk, the sedimentation velocity was calculated as follows:

$$v=\frac{g\left(\rho_{1}-\rho_{2}\right)d^{2}}{18\eta },$$

In the above formula, *v* is the sedimentation velocity, nm/s; *g* is the gravitational acceleration, 9.80 m/s^2^; *ρ*_1_ represents the particle density (density of all particles except water in soymilk), g/cm^3^; *ρ*_2_ denotes the water density, g/cm^3^; *d* refers to the particle diameter, cm; 18 is the conversion coefficient; and *η* is the viscosity of water, Pa·s.

### 2.7 Predictive model of soymilk shelf‐life

The changes in soymilk stability indices during storage showed zero‐ or first‐order kinetics (Wu et al., 2021). Temperature is an important factor affecting the reaction rate. Therefore, the kinetic model combined with the Arrhenius equation was used to predict the shelf‐life of soymilk at different storage temperatures (Singh et al., 2021).

If the reaction is a zero‐order reaction, the reaction rate constant is calculated as follows:

$$C_{0}-C=\mathrm{kt}$$

If the reaction is a first‐order reaction, the reaction rate constant of the reaction is calculated as follows:

$$\ln\left(C_{0}/C\right)=\mathrm{kt}$$

In the above equations, *C*_0_ is the initial value of the stability parameter; *C* is the stability parameter of time *t*; and *k* is the reaction rate constant.

The reaction rate constant depends on the reaction temperature and can be expressed by the following Arrhenius equation:

$$k=A_{0}\exp\left(-E_{a}/\mathrm{RT}\right)$$

In the above formula, *A*_0_ is the Arrhenius constant; *E*_a_ is the activation energy (kJ/mol); *R* refers to the absolute gas constant 8.314 (J/mol·K); and *T* represents the absolute temperature K.

In order to further understand the kinetics and thermodynamic reaction mechanism of soymilk during storage, according to the absolute reaction rate theory, the thermodynamic parameters ΔH*, ΔS*, and ΔG* of the reaction can be calculated as follows:

$${\mathrm{\Delta H}}^{*}=E_{a}-\mathrm{RT}$$

$${\mathrm{\Delta S}}^{*}=R\left[\ln K-\ln\left(k_{b}/h\right)-\ln T-1\right]+E_{a}/T$$

$$\Delta G^{*}=\Delta H^{*}-T\Delta S^{*}$$

In the above formula, *H* is the enthalpy; *S* represents the entropy; *G* denotes Gibbs free energy; *E*_a_ refers to the activation energy (kJ/mol); *R* indicates the absolute gas constant 8.314 (J/mol·K); *T* is the absolute temperature K; *k*_b_ is the Boltzmann constant (1.38 × 10^−23^ J/K); and *h* is the Planck's constant (6. 626 × 10^−34^ J/s).

### 2.8 Statistical analysis

Data are presented as the mean ± standard deviation (SD). SPSS 22.0 and origin 10.0 were used for statistical analysis and analysis of variance (ANOVA). Significant and extremely significant levels were set at *p* < .05 to identify statistically significant differences among groups.

## 3 RESULTS AND DISCUSSION

### 3.1 Effects of different processing methods on the nutritional composition of soymilk

**FIGURE 2:**
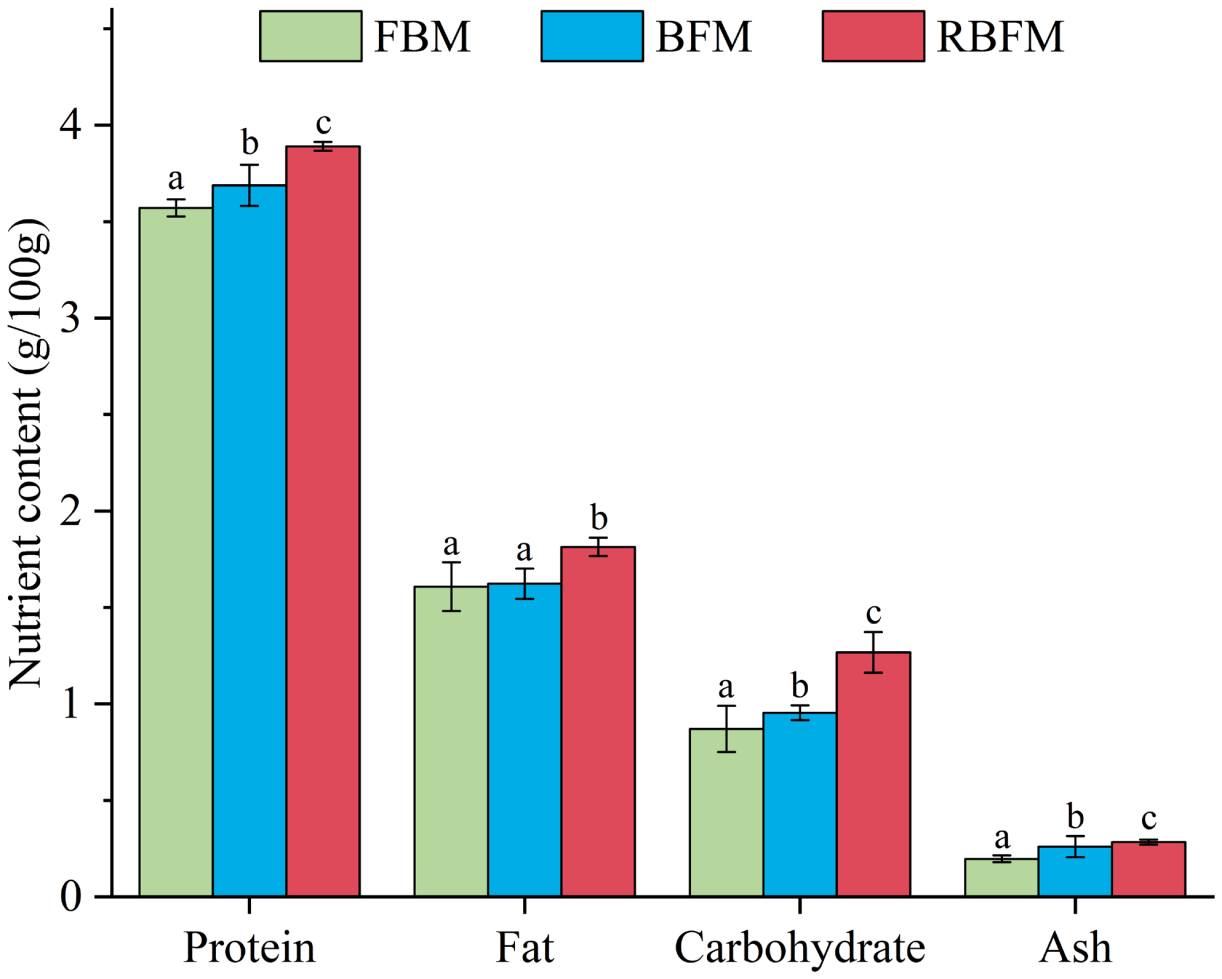
Effects of different processing methods on the nutritional composition of soymilk. WHC refers to water holding capacity. The different lowercase letters above the columns represent treatment groups that are significantly different from each other (*p* < .05). BFM, boiling‐to‐filtering method; FBM, filtering‐to‐boiling method; RBFM, repeated boiling‐to‐filtering method.

The changes in nutritional composition of soymilk under different processing methods are shown in Figure 2. Soymilk produced by RBFM has the highest nutrient extraction rate, with a protein content of 3.89 g/100 g, a fat content of 1.81 g/100 g, and a carbohydrate content of 1.27 g/100 g. The soymilk produced by BFM yielded the second highest nutrients (proteins, 3.69 g/100 g; fat, 1.62 g/100 g; and carbohydrates, 0.954 g/100 g). The nutrient extraction rate was the least with FBM, resulting in a protein content of 3.57 g/100 g, fat content of 1.61 g/100 g, and carbohydrate content of 0.87 g/100 g, probably due to the incomplete release of soybean nutrients into soybean homogenate.

The trends in nutritional composition of soymilk under different processing technologies were highly similar, consistent with the results of Fan et al. (2020). When soymilk is filtered and heated, several nutrients are separated along with the soybean dregs, resulting in fewer nutrients being released in FBM than in the other two methods (Kyoko et al., 2007; Li et al., 2021). In addition, the soybean dregs were washed and extracted into soybean homogenate by repeated heating in RBFM, and the soybean homogenate was repeatedly heated to dissolve more protein, fat, soluble polysaccharides, and other nutrients, resulting in soymilk with the highest nutrient levels (Fan et al., 2020; James & Yang, 2016).

### 3.2 Effects of different processing methods on the physical properties of soymilk

**FIGURE 3:**
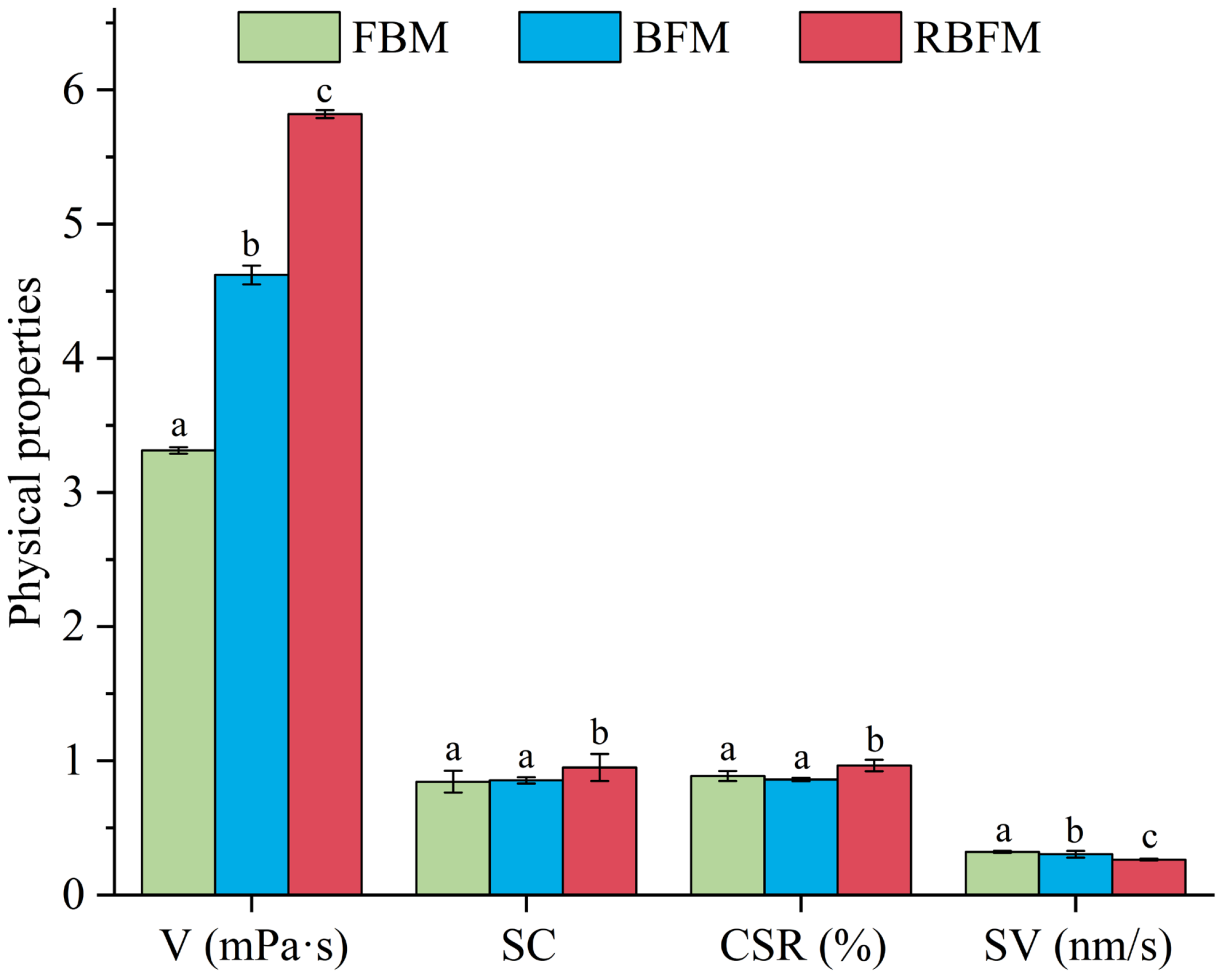
Effects of different processing methods on the physical properties of soymilk. V, SC, CSR, and SV represent viscosity, stability coefficient, centrifugal sedimentation rate, and sedimentation velocity, respectively. The different lowercase letters above the columns refer to treatment groups that are significantly different from each other (*p* < .05). BFM, boiling‐to‐filtering method; FBM, filtering‐to‐boiling method; RBFM, repeated boiling‐to‐filtering method.

The changes in the physical properties of soymilk under different processing methods are shown in Figure 3. The viscosity, stability coefficient, and CDR of soymilk in the RBFM group were significantly higher than in the BFM and FBM groups (*p* < .05), and reached maximum values of 5.82, 0.950, and 0.965, respectively. Generally, the stability coefficient is ≤1. The larger the stability coefficient, the more stable the soymilk system is, which indicates that RBFM soymilk is more uniform and stable (Pires et al., 2003; Yang et al., 2016). Yu et al. (2015) and Huang et al. (2022) have reported that the RBFM may facilitate the dissolution of polysaccharides and phospholipids, and thus ensure that solid dispersions and liquid emulsions form a solid and stable multi‐component complex with good emulsification characteristics. It not only effectively prevents fat polymerization and the formation of large floating oil bodies but also prevents protein precipitation, thus enhancing the stability and CDR of soymilk (Fan et al., 2020; Huang, He, et al., 2021). The nutrients in soymilk generated by BFM and FBM mostly exist in free form and do not form a stable structure, resulting in poor stability and lower CDR. This result has been confirmed in a previous report (Wu et al., 2014).

During dairy processing, product stability can be determined by analyzing the changes in dairy sedimentation velocity. Thus, the factors contributing to product instability during production can be identified (Amine et al., 2022; Chaturvedi et al., 2019). The particle and medium density of soybean products do not change significantly. The viscosity and particle size of soymilk are the two main factors affecting the sedimentation rate (Fan et al., 2020; Miguel et al., 2022; Zhang et al., 2008). In this study, the sedimentation rate of soymilk processed via RBFM was significantly lower (0.264 nm/s) than that of soymilk obtained via BFM (0.304 nm/s) and FBM (0.321 nm/s; *p* < .05). The dissolution rate of soluble solids in RBFM soymilk was higher, which increased the viscosity of soymilk and reduced its sedimentation rate (Fan et al., 2020; Yuya et al., 2021). However, repeated heating increased the distribution of acidic polypeptide and 7S subunits α and α′ on the surface of protein particles and improved the hydrophilicity of protein particles (Gong et al., 2023), thereby increasing the viscosity of soymilk and reducing its sedimentation rate. Based on the changes in nutrient content and physical properties of soymilk obtained via different methods, the soymilk generated via RBFM had higher nutrient levels and more stable physical properties, so it was selected to study the storage stability and predict the shelf‐life.

### 3.3 Effects of different storage temperatures on sensory scores and storage stability of soymilk processed via RBFM

**FIGURE 4:**
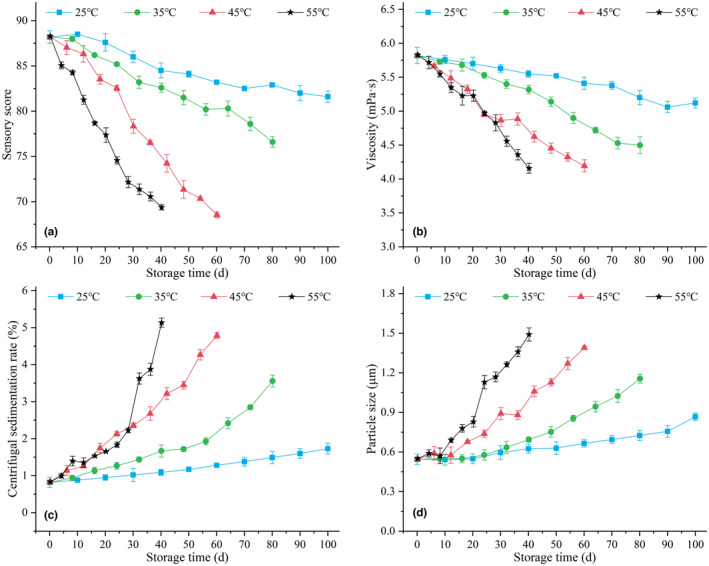
Changes in sensory score and storage stability of soymilk obtained via RBFM at different storage temperatures. (a–d) indicate the sensory score, viscosity, centrifugal sedimentation rate, and particle size, respectively.

The changes in sensory scores of soymilk processed via RBFM during storage at different temperatures are shown in Figure 4a. The sensory scores of soymilk under different storage temperatures declined, and the sensory scores of soymilk decreased by 6.6, 11.6, 19.7, and 18.9 at 25°C, 35°C, 45°C, and 55°C, respectively, indicating that the higher the storage temperature, the lower the sensory scores of soymilk. Compared with soymilk at 25°C, the sensory deterioration at 45°C and 55°C was more significant, and the sensory scores dropped below 80 on days 30 and 20, respectively. The balance between protein and fat levels in soymilk was destroyed during high‐temperature storage (Li, He, et al., 2022; Li, Wan, et al., 2022), which led to a decline in soymilk texture. However, the fat in soymilk may be oxidized to generate bad flavor (Wang et al., 2021), resulting in a reduced overall sensory score.

The changes in the viscosity of soymilk obtained via RBFM during storage at different temperatures are shown in Figure 4b. The viscosity of soymilk at different storage temperatures showed a decreasing trend. At the end of storage at 25°C, 35°C, 45°C, and 55°C, the viscosity of soymilk decreased by 0.70, 1.32, 1.63, and 1.67 mPa·s, respectively, indicating that the viscosity of soymilk declined with the increase in storage temperature and storage time. The prolonged storage may degrade the macromolecular substances such as pectin (acting as a stabilizer) and soybean lecithin (acting as an emulsifier) in the soymilk (Ding et al., 2020; Wang et al., 2013), which reduced internal friction and thus decreased the viscosity of the soymilk.

The changes in CSR of soymilk obtained via RBFM during storage at different temperatures are shown in Figure 4c. At a storage temperature of 25°C, the CSR of RBFM soymilk showed a slightly upward trend with the extension of time, ranging from 0.82 to 1.73. At storage temperatures of 35°C, 45°C, and 55°C, the CSR of soymilk increased significantly with storage time, from the initial value of 0.82% to the highest values of 3.56%, 4.78%, and 5.12%, respectively, indicating that the higher the storage temperature, the greater the increase in the CSR of soymilk. It is speculated that with the increase in time and temperature, the protein molecules in the soymilk collide with each other, resulting in particle aggregation and an increased particle size of the soymilk (Li, He, et al., 2022; Li, Wan, et al., 2022; Wang et al., 2018). In addition, the higher the temperature, the higher the collision frequency between particles. High temperatures also increase protein denaturation, which raises the CSR of soymilk.

The changes in particle size of soymilk produced by RBFM during storage at different temperatures are shown in Figure 4d. The changes in soymilk particle size and CSR were basically identical during storage at different temperatures. At a storage temperature of 25°C, the particle size of soymilk increased slightly with time, and increased to a maximum of 0.322 μm at the end of storage time. Under the storage temperatures of 35°C, 45°C, and 55°C, the particle size of soymilk increased significantly with time, from the initial value of 0.545 μm to the maximum value of 1.156, 1.387, and 1.486 μm, respectively, indicating that the particle size of soymilk increased gradually with the increase in storage temperature and storage time. The protein and fat stability of soymilk may be destroyed during high‐temperature storage, which causes the aggregation or fusion of free fat globules and protein particles in soymilk (Peng et al., 2016), leading to an increase in particle size. However, the charges and potential energies between proteins, fats, carbohydrates, and other macromolecules in soymilk change, resulting in particle collision or aggregation (Huang et al., 2022; Lu et al., 2013). Higher temperatures increase the collision frequency and aggregation, which increase soymilk particle size and volume.

### 3.4 Reaction thermodynamics and kinetic parameters of storage stability indices of soymilk processed via RBFM

In this study, soymilk during storage exhibits first‐order reaction kinetics. A regression analysis of CDR, viscosity, and particle size of soymilk during storage at different temperatures was conducted (Jia et al., 2020; Singh et al., 2009), and their reaction rate constant (*k*), reaction activation energy (*E*_*a*_), and reaction thermodynamic parameters (ΔH*, ΔS*, and ΔG*) were calculated (Table 2).

**TABLE 2:** Kinetics and thermodynamic parameters of soymilk stability during storage.

Stability indexes	Temperature (K)	*k* (d^−1^)	*E*_*a*_ (KJ/Mol)	ΔH* (KJ/Mol)	ΔS* (J/Mol·K)	ΔG* (KJ/Mol)
Centrifugal sedimentation rate	298.15	0.0367	40.8	38.32	−143.85	81.21
	308.15	0.0678	40.8	38.24	−143.47	82.45
	318.15	0.1013	40.8	38.15	−144.56	84.15
Particle size	298.15	0.0158	38.58	36.10	−158.31	83.30
	308.15	0.0270	38.58	36.02	−158.33	84.81
	318.15	0.0420	38.56	35.91	−158.92	86.47
Viscosity	298.15	0.0630	10.56	8.08	−240.79	79.87
	308.15	0.0112	10.56	8.00	−256.57	87.06
	318.15	0.0168	10.56	7.91	−254.54	88.90

*E*_*a*_ refers to the energy required for a molecule to transition from a normal state to an active state. The lower *E*_*a*_, the easier it is for chemical reactions to occur (Singh et al., 2009, 2021). The *E*_*a*_ of viscosity is the lowest, which may be due to the destruction of the stable structure of soymilk, reduced particle size, and decreased shear force and friction force between the two layers of fluid during storage (Luo, 2009; Zhang et al., 2017), leading to significant changes in viscosity. The ΔH* of soymilk at different temperatures during storage was greater than zero, indicating that the first‐order kinetic reaction of soymilk was an endothermic reaction. Higher temperatures promoted the formation of complexes and adversely affected the stability of the soymilk system (Zhang et al., 2008). Gibbs free energy (ΔG*) is the component of the reduced internal energy of the system that can be converted into external work in a thermodynamic process. When ΔG* > 0, it is a non‐spontaneous reaction, and when ΔG* < 0, it is a spontaneous reaction (Vikraman et al., 2021). In this study, the ΔG* of soymilk during storage ranged from 79.87 to 88.90 kJ/mol, indicating non‐spontaneous reactions of soymilk during storage at different temperatures.

In addition, the ΔG* of soymilk during storage at different temperatures gradually increased with the increase in temperature, so the regression equation between ΔG* and temperatures can be established to reveal the correlation between chemical reactions and macro‐physical changes. The regression equation between ΔG* of each stability index of soymilk and temperature during storage at different temperatures is presented in Table 3. The correlation coefficient of the equation was greater than 0.89, indicating that these equations can be used to predict the ΔG* of soymilk stability at different temperatures.

**TABLE 3:** Gibbs free energy and temperature regression equation of storage stability of soymilk.

Stability indexes	Regression equation ΔG* (KJ/Mol) = *f*(*T*)	*R* ^2^
Centrifugal sedimentation rate	ΔG* = 0.1467 *T* + 37.398	.9918
Particle size	ΔG* = 0.1587 *T* + 35.964	.9991
Viscosity	ΔG* = 0.4513 *T* ‐ 53.788	.8951

### 3.5 Evaluation of RBFM soymilk shelf‐life

#### 3.5.1 Establishment of an RBFM soymilk shelf‐life prediction model

Sensory evaluation is generally used to evaluate the quality changes in soymilk (Albisu et al., 2023). In this study, the storage stability index with a high degree of correlation with the sensory score was used as the evaluation parameter to establish the shelf‐life prediction model of soymilk. The sensory score of soymilk was correlated with CSR, viscosity, and particle size using SPSS 22.0. The higher the correlation coefficient of the regression equation, the more accurate the prediction model was, and the closer the prediction result was to the actual value (Albisu et al., 2023; Singh et al., 2021). The correlation between storage stability indices and sensory scores of soymilk during storage is shown in Table 4. The correlation coefficient between the CSR of soymilk and the sensory score was the highest (*R* ^2^ = .9868), indicating the strongest correlation.

**TABLE 4:** Correlation between storage stability indexes and sensory scores of soymilk.

Indexes	Regression equation	*R* ^2^
Sensory score and centrifugal sedimentation rate (CSR)	SS = −9.6927CSR + 96.131	.9868
Sensory score and particle size (*d*)	SS = −33.187*d* + 105.79	.9796
Sensory score and viscosity (*η*)	SS = 10.322*η* ‐ 28.007	.9558

Therefore, the CSR was selected as the key indicator to predict the shelf‐life of soymilk. Based on the change of CSR in soymilk during storage, a prediction model for the shelf‐life of soymilk was established, according to the following equation:

$$\mathrm{SS}=-9.6927\left\{{\mathrm{CSR}}_{0}\mathrm{esp}\left[t\frac{k_{b}T}{h}\mathrm{esp}\frac{-\left(146.7\times T+37398\right)}{\mathrm{RT}}\right]\right\}+96.131$$

In the above equation, *CSR*_*0*_ is the initial centrifugal precipitation rate; *k*_*b*_ is the Boltzmann constant (1.38 × 10^−23^ J/K); *t* denotes the storage time; *h* represents the Planck's constant (6. 626 × 10^−34^ J/s); *R* is the absolute gas constant (8314 J/mol·K); and *T* is the absolute temperature K.

#### 3.5.2 Evaluation of the RBFM soymilk shelf‐life prediction model

The accuracy of the soymilk shelf‐life prediction model can be tested based on the standard error (*S*_*e*_) between the predicted value of the model and the actual value. In addition, the residual variation coefficient (*V*_*e*_) of the prediction model represents the percentage ratio of the *S*_*e*_ of the prediction model to the average measured value (Jia et al., 2020; Wu et al., 2021). *S*_*e*_ and *V*_*e*_ can be calculated using the following formula:

$$S_{e}=\sqrt{\frac{\sum_{i=1}^{n}{\left(y_{i}-{\hat{y}}_{i}\right)}^{2}}{n-k-1}}$$

$$V_{e}=\frac{S_{e}}{\bar{y}}\times 100\%$$

In the above formulas, *S*_*e*_ is the standard error of prediction; ${\hat{y}}_{i}$ is the predicted value of the model; $y_{i}$ denotes the measured value of the model; $\bar{y}$ represents the average measured value; *n* is the sample number; and *k* is the number of prediction factors.

In order to verify the accuracy of the soymilk shelf‐life prediction model, the observed and predicted values were compared and evaluated at 25°C, 35°C, and 45°C. In beverages and dairy products, the *V*_*e*_ is less than 15%, indicating that the accuracy of the predictive model meets the requirements (Wu et al., 2021; Wunderlich et al., 2023). In this study, the observed stability times of soymilk samples were 90, 120, and 150 days, and the *V*_*e*_ of the prediction model was 10.78% (less than 15%), indicating that the accuracy of the model was good and met the requirements for predicting the shelf‐life of soymilk.

## 4 CONCLUSIONS

This study investigated the effects of different processing methods on the quality and nutrition of soymilk, as well as the storage stability and shelf‐life of soymilk at different storage temperatures. The results suggest that soymilk processed via RBFM exhibited the highest nutrient content and physical properties. The sensory score and stability indices of soymilk processed via RBFM changed significantly during storage at different temperatures, and the correlation coefficient between the CSR and the sensory score was the highest (*R* ^2^ = .9868). A shelf‐life prediction model was constructed with CSR as the key indicator to evaluate soymilk processed via RBFM. The *V*_*e*_ of the predictive model was 10.78% (less than 15%), indicating that the accuracy of the model was good and can be used to predict the shelf‐life of soymilk processed via RBFM and stored at 25–45°C. However, further studies (involving, for example, sensory quality, flavor, and nutrients) are required to verify the predictive mode of soymilk and provide an effective theoretical basis for the development, transportation, and storage of soymilk.

## AUTHOR CONTRIBUTIONS

**Liu Fan:** Methodology (equal); writing – original draft (equal). **Yitong Duan:** Methodology (equal); software (equal). **Zhanrui Huang:** Conceptualization (equal); writing – review and editing (equal). **Dan Zhao:** Methodology (equal); writing – review and editing (equal). **Liangzhong Zhao:** Conceptualization (equal); writing – review and editing (equal). **Wanying He:** Validation (equal); writing – original draft (equal). **Xuejiao Zhang:** Investigation (equal); validation (equal). **Ming Li:** Investigation (equal); resources (equal). **Yingyi Lin:** Resources (equal). **Yu Chen:** Resources (equal).

## FUNDING INFORMATION

This work was supported by the Science and Technology Innovative Program of Hunan Province (No. 2019TP1028), the Regional Joint Funds of Hunan Provincial Natural Science Foundation of China (No. 2022JJ50232), and the project of the Hunan Education Department (21A0480).

## CONFLICT OF INTEREST STATEMENT

The authors declare that they have no conflict of interest in this study.

## Data Availability

Research data are not shared.
